# Case Report: A diagnostic pitfall of diffuse large B-cell lymphoma with hemophagocytic lymphohistiocytosis and atypical tongue pain: the critical role of PET/CT-guided repeat bone marrow biopsy

**DOI:** 10.3389/fonc.2026.1804997

**Published:** 2026-04-10

**Authors:** Lingpeng Meng, Jiayue Li, Ran Chen, Xinlei Wang, Yantong Zhuge, Qingyin Kong, Qipeng Jin, Yiyi Zhang

**Affiliations:** Department of Critical Care Medicine, Longhua Hospital, Shanghai University of Traditional Chinese Medicine, Shanghai, China

**Keywords:** bone marrow, diffuse large B-cell lymphoma, fever of unknown origin, hemophagocytic lymphohistiocytosis, positronemission tomography

## Abstract

**Background:**

Diffuse large B-cell lymphoma (DLBCL) associated hemophagocytic lymphohistiocytosis (HLH) represents a rare but life-threatening clinical entity characterized by diagnostic complexity and high mortality. Early manifestations are often nonspecific, and initial bone marrow examination may be falsely negative due to patchy infiltration, leading to delayed recognition of the underlying malignancy.

**Case presentation:**

We report a 68-year-old woman who presented with prolonged fever and atypical tongue pain, initially suspected to be caused by pulmonary infection. Despite broad-spectrum antimicrobial therapy, fever persisted and progressive cytopenias, hyperferritinemia, hypofibrinogenemia, and elevated soluble interleukin-2 receptor levels fulfilled the HLH-2004 diagnostic criteria. Although corticosteroid therapy achieved transient clinical improvement, the initial bone marrow biopsy revealed no malignancy. Following relapse of fever after steroid withdrawal, positron emission tomography-computed tomography (PET/CT) demonstrated diffuse hypermetabolic activity in the spleen and bone marrow. PET/CT-guided repeat bone marrow biopsy subsequently confirmed non-germinal center B-cell-type DLBCL with marrow involvement. The patient achieved clinical remission after initiation of immunochemotherapy.

**Conclusion:**

This case highlights HLH as a major diagnostic blind spot in occult B-cell lymphoma and underscores the limitations of single-site bone marrow biopsy. Persistent atypical extranodal symptoms such as tongue pain, together with steroid-responsive HLH, should prompt early functional imaging. PET/CT-guided repeat tissue sampling plays a pivotal role in establishing timely diagnosis and guiding therapeutic decisions in critically ill patients.

## 1 Introduction

Diffuse large B-cell lymphoma (DLBCL) is one of the most common subtypes of non-Hodgkin lymphoma (NHL), accounting for approximately 30%-40% of all newly diagnosed cases of NHL in adults. It is characterized by significant biological heterogeneity and can involve both lymph nodes and various extranodal sites, with diverse clinical features, morphology, and genetic abnormalities (1). Accurate diagnosis and timely targeted therapy are crucial in the management of this disease. Early diagnosis and intervention have been shown to significantly improve survival outcomes for DLBCL patients (2).

In the head and neck region, DLBCL most commonly involves the maxilla, mandible, palatal soft tissues, oral vestibule, or gingiva, whereas involvement of the tongue is distinctly uncommon. Available case series and sporadic reports indicate that when the tongue is affected, clinical manifestations are often nonspecific, including localized pain, swelling, or mucosal discomfort, and may occur in the absence of an obvious mass lesion or palpable lymphadenopathy. The tongue possesses a complex lymphatic drainage network and lies in close anatomical proximity to surrounding neuromuscular structures. Consequently, minimal infiltration of the submucosal or muscular tissues, or irritation of adjacent neural structures, may give rise to early sensory symptoms without clear localizing signs on routine examination. Under such circumstances, tongue pain is more likely to be attributed to infectious or inflammatory conditions, particularly when accompanied by systemic inflammatory responses, which may further obscure the recognition of an underlying hematologic malignancy (3–6).

Hemophagocytic Lymphohistiocytosis (HLH) is a life-threatening syndrome caused by excessive activation of the immune system. It is characterized by fever, elevated ferritin and other systemic inflammatory markers, abnormal blood cell counts, disseminated intravascular coagulation, hepatosplenomegaly, central nervous system (CNS) inflammation, and multi-organ dysfunction, leading to shock and high mortality. HLH can be triggered by tumors, infections, or autoimmune diseases. Among these, lymphoma-associated HLH(LAHS) is the most common, although HLH secondary to B-cell lymphoma is relatively rare. The clinical presentation often mimics that of infectious diseases, leading to diagnostic confusion (7–9). This case highlights the diagnostic challenges of HLH when it presents as the initial manifestation of B-cell lymphoma and emphasizes the importance of timely identification of B-cell Lymphoma-Associated HLH(B-cell LAHS) for early treatment and improved prognosis.

Positron Emission Tomography-Computed Tomography(PET/CT), as a non-invasive diagnostic tool that combines PET imaging and radiology, effectively reveals disease radiological patterns that may be overlooked by traditional imaging techniques, especially in the diagnosis of bone marrow involvement (10). In cases of DLBCL, particularly with focal bone marrow involvement, PET/CT demonstrates high accuracy and significantly improves the diagnostic positivity rate (11).

This report describes a case of B-cell lymphoma in which prolonged fever of unknown origin accompanied by tongue pain constituted the initial clinical presentation. Infectious etiologies were primarily considered at admission; however, antimicrobial therapy was ineffective, and as the disease progressed, the patient gradually developed clinical and laboratory abnormalities fulfilling the diagnostic criteria for HLH. The initial bone marrow biopsy was negative, and neither immunohistochemistry nor flow cytometry yielded a definitive diagnosis. Ultimately, following PET/CT findings suggestive of an underlying hematologic malignancy, a second bone marrow biopsy confirmed the diagnosis of DLBCL.

This case highlights the diagnostic challenge posed by the temporal dissociation between early presenting symptoms and subsequent HLH manifestations in B-cell lymphoma and underscores the pivotal role of PET/CT in the evaluation of such complex cases. Persistent atypical extranodal symptoms, such as tongue pain, should prompt heightened clinical vigilance to facilitate early identification of underlying lymphoma or other malignancies.

## 2 Case presentation

A 68-year-old woman with a 30-year history of well-controlled hypertension presented with recurrent fever lasting over six months, accompanied by progressive cough, sputum production, dyspnea, and intermittent tongue pain over the preceding month. She denied night sweats, weight loss, or palpable lymphadenopathy.

On admission, her temperature was 38.0°C, blood pressure 106/67 mmHg, heart rate 82 beats/min, respiratory rate 20 breaths/min, and oxygen saturation 98% on 4 L/min nasal oxygen. Physical examination revealed coarse bilateral breath sounds with scattered moist rales. No hepatosplenomegaly or superficial lymphadenopathy was detected, and oral examination showed no visible ulceration or mass lesion.

Initial laboratory testing demonstrated leukopenia (3.66 × 10^9^/L), anemia (hemoglobin 99g/L), and elevated C-reactive protein (33mg/L) (**Table 1**). Chest computed tomography revealed patchy infiltrates and consolidation in both lungs, consistent with community-acquired pneumonia. Empirical antimicrobial therapy with moxifloxacin was initiated.

**Table 1:** Auxiliary examinations upon admission.

Test	Date	Result
Complete Blood Count (CBC)	2025/6/30	WBC: 3.66×10^9^/L, HB: 99 g/L, PLT: 165×10^9^/L, N: 55%, L: 1.3×10^9^/L
C-Reactive Protein (CRP)	2025/6/30	33 mg/L
Procalcitonin (PCT)	2025/6/30	0.41 ng/mL
EB Test	2025/6/30	Negative
Sputum PCR	2025/6/30	Negative
Tuberculosis T-cell Infection Test	2025/6/30	Negative
Chest CT	2025/6/30	Right upper lobe and horizontal fissure solid nodule, indicating pulmonary infection
Abdominal Ultrasound	2025/6/30	Fatty liver, hepatic cyst, left renal stone
Echocardiogram	2025/6/30	EF: 79%, SV: 66 mL, moderate aortic valve regurgitation, mild mitral, tricuspid, and pulmonary valve regurgitation, left ventricular diastolic dysfunction
Lower Extremity Doppler Ultrasound	2025/6/30	Bilateral femoral artery plaques detected
Electrocardiogram (ECG)	2025/6/30	No significant abnormalities

**Figure 1:**
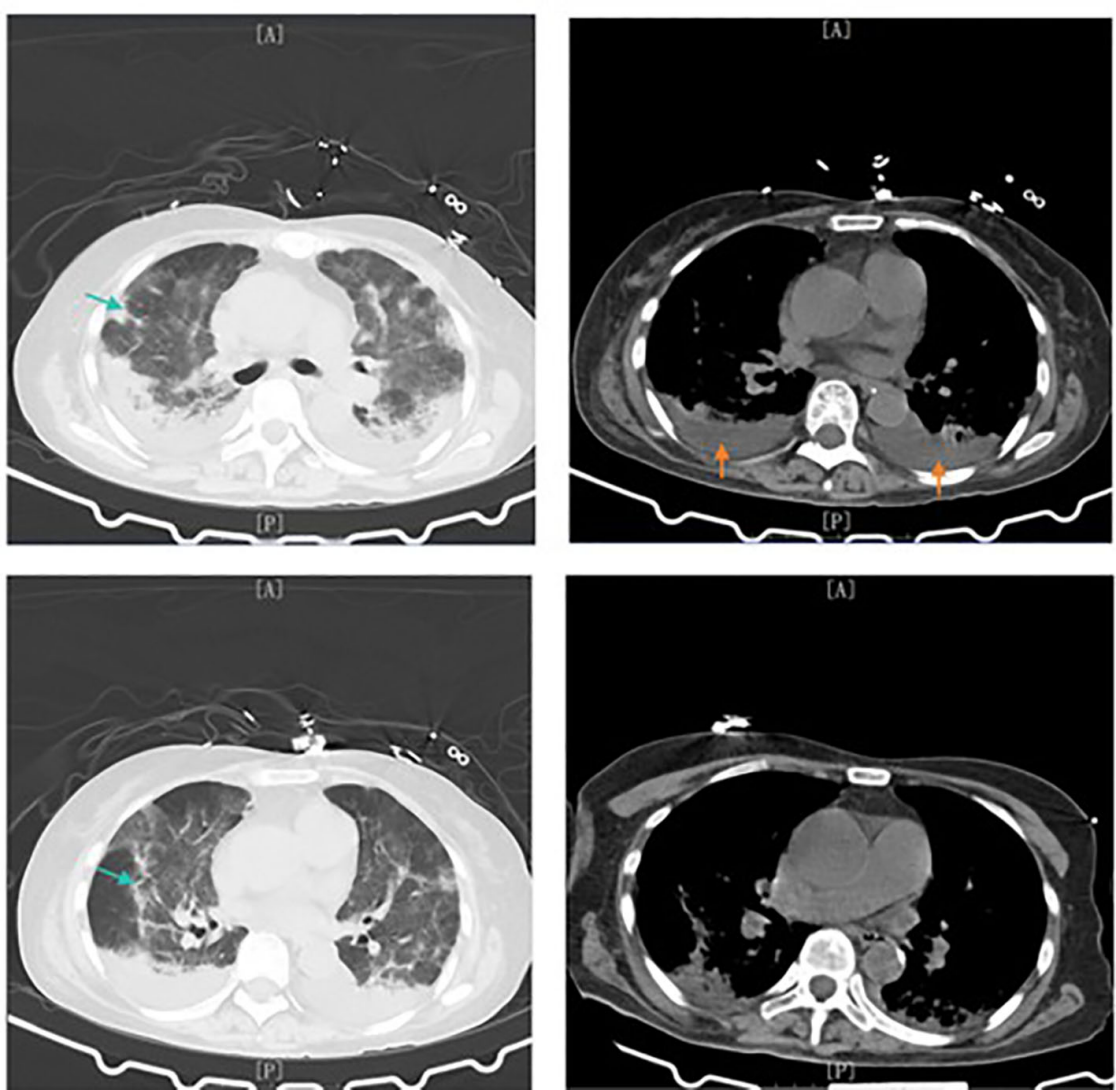
Initial Chest Computed Tomography Findings Chest CT obtained on July 2, 2025 demonstrates scattered bilateral patchy opacities with ill-defined margins and partial consolidation, consistent with pulmonary infection. Green arrows indicate interstitial infiltrates and diffuse inflammatory changes, while orange arrows denote bilateral pleural effusions.

Despite broad-spectrum antibiotics, the patient developed persistent high-grade fever (up to 39.2 °C), worsening hypoxemia, and progressive respiratory distress, necessitating transfer to the intensive care unit. Repeat chest CT demonstrated diffuse interstitial infiltrates with bilateral pleural effusions, raising concern for acute respiratory distress syndrome (**Figure 1**). High-flow nasal oxygen was commenced, and antimicrobial coverage was escalated to piperacillin-tazobactam, vancomycin, and levofloxacin. Sputum next-generation sequencing detected *Rhodococcus equi*, while blood microbial sequencing remained negative.

Chest CT obtained on July 2, 2025 demonstrates scattered bilateral patchy opacities with ill-defined margins and partial consolidation, consistent with pulmonary infection. Green arrows indicate interstitial infiltrates and diffuse inflammatory changes, while orange arrows denote bilateral pleural effusions.

Although pulmonary infiltrates gradually improved with antimicrobial therapy, fever persisted and was accompanied by progressive cytopenias, hyperferritinemia (>2000 ng/mL), hypofibrinogenemia, and markedly elevated soluble interleukin-2 receptor levels (>7500 U/mL). These findings fulfilled the HLH-2004 diagnostic criteria, prompting consideration of secondary HLH (**Table 2** and **Supplementary Table 1**).

**Table 2:** Auxiliary examinations during hospitalization.

Parameter	Day 1 (Jul 1)	Day 4 (Jul 4)	Day 6 (Jul 6)	Day 8 (Jul 8)	Day 10 (Jul 10)	Day 16 (Jul 16)
WBC(×10^9^L)	5.71	4.36	2.27	1.23	4.07	2.25
Neutrophils(×10^9^L)	3.03	1.36	0.25	0.07	2.53	1.53
Lymphocytes(×10^9^L)	2.07	2.09	1.49	0.53	0.72	0.5
Monocytes(×10^9^L)	0.59	0.88	0.52	0.61	0.55	0.17
Hemoglobin(g/L)	78	74	85	86	82	97
Platelets(×10^9^L)	126	132	191	171	187	81
CRP(mg/L)	47.71	38	41.76	35.2	–	13.02
PCT(ng/mL)	0.961	0.493	0.099	–	–	5.255
Triglycerides(mmol/L)	–	–	–	1.82	–	–
Ferritin(ng/ml)	–	–	–	–	–	>2000
Fibrinogen(g/L)	–	2.4	2.7	1.6	1.2	1.6
SIL-2R(U/ml)	–	–	–	>7500	–	>7500

**Figure 2:**
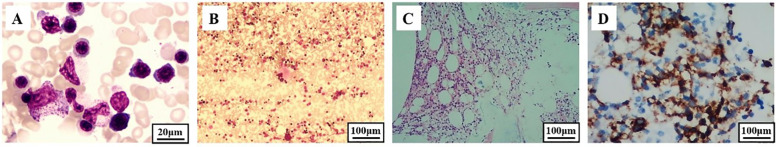
Initial bone marrow examination. **(A)** High-magnification bone marrow smear (Wright-Giemsa stain) showing representative hematopoietic cells with trilineage hematopoiesis. **(B)** Low-magnification bone marrow smear demonstrating active hematopoiesis and overall cellular distribution. **(C)** Bone marrow biopsy section with hematoxylin and eosin staining showing active hematopoiesis without evidence of lymphoid infiltration. **(D)** Immunohistochemical staining (CD71) highlighting erythroid precursors in the bone marrow. Overall, the initial bone marrow examination showed reactive hematopoietic changes without definitive evidence of hematologic malignancy. Scale bars: 20 μm **(A)**; 100 μm **(B–D)**.

A unilateral bone marrow aspiration and biopsy were performed at the right anterior superior iliac spine. The biopsy specimen measured approximately 0.6 × 0.2 × 0.2 cm and was considered technically adequate for histopathologic evaluation. Morphological examination revealed a hypercellular marrow with active trilineage hematopoiesis and an inverted myeloid-to-erythroid ratio. No excess blasts, atypical lymphoid aggregates, or plasma cell proliferation were identified. Importantly, no morphologic evidence of malignancy or hemophagocytosis was observed (**Figure 2** and **Table 3**).

**Table 3:** Comparison of three bone marrow examinations during the diagnostic course.

Parameter	First bone marrow examination	Second bone marrow examination (DLBCL diagnosed)	Third bone marrow examination (Follow-up)
Puncture site	Right anterior superior iliac spine	Right posterior superior iliac spine	Right posterior superior iliac spine
Specimen size	One core biopsy, 0.6 × 0.2 × 0.2 cm	Two gray-brown tissue fragments, 0.2-0.3 × 0.2 cm	Three gray-white fragments, 0.2-0.4 cm
Marrow cellularity	Hypercellular marrow (approximately 45% nucleated cells)	Hematopoietic components occupying ~50% of trabecular space	Hematopoietic components occupying ~30% of trabecular space
Myeloid-to-erythroid ratio	Inverted	Not reported	1.24: 1
Granulocytic lineage	All maturation stages present, predominantly mature cells	Granulocytic cells present	Hyperplasia with all maturation stages present
Erythroid lineage	All maturation stages present, mainly mid-late erythroblasts	Erythroid cells present	Markedly active erythropoiesis with nuclear division observed
Megakaryocytic lineage	Normal number, scattered distribution	Megakaryocytes present	Hyperplasia with platelets scattered or clustered
Lymphoid cells	No significant increase or aggregation of lymphocytes or plasma cells	Sheets of atypical large lymphoid cells observed	Mature lymphocytes 10.5%; scattered T and B lymphocytes
Immunohistochemistry	CD34(+), CD117(+), CD42b(+), CD3(+ few), CD20(+ few), CD138(+ few), CD71(+), CKpan(−)	Gly(+), MPO(+), CD61(+), CD20(+), CD79a(+), CD3(+ few), CD117(scattered+), CD34(−)	CD61(+), CD31(+), CD69(+), CD34(−), CD79a(+), MUM1(scattered+), CD3(scattered+), PAX5(scattered+)
DLBCL-related markers	Not performed	Bcl-2(+), Bcl-6(−), CD10(−), MUM1(partial+), C-MYC (~30%+), CD79b(focal+)	Not performed
Flow cytometry	No immunophenotypic evidence of leukemia, lymphoma, or myeloma; polyclonal B cells (~8.3% of lymphocytes); CD34+ progenitors ~0.3%	Monoclonal B-cell population (~1.3% of total cells), CD5+, CD10−	Not performed
EBV study	Not performed	EBER negative	Not performed
Special staining	Reticulin stain: MF-0	Reticulin (0), PAS (+)	Reticulin (−), PAS (+)
Pathological interpretation	Hypercellular marrow without specific morphologic features of lymphoma	Bone marrow involvement by diffuse large B-cell lymphoma (non-GCB type)	No morphologic evidence of lymphoma involvement

Given the strong clinical suspicion of HLH, corticosteroids combined with intravenous immunoglobulin were initiated, resulting in transient defervescence and partial relief of tongue pain. However, following tapering of corticosteroids, the patient experienced recurrence of high-grade fever (up to 40.0 °C) and worsening systemic symptoms, despite radiologic resolution of pulmonary infection.

**Figure 3:**
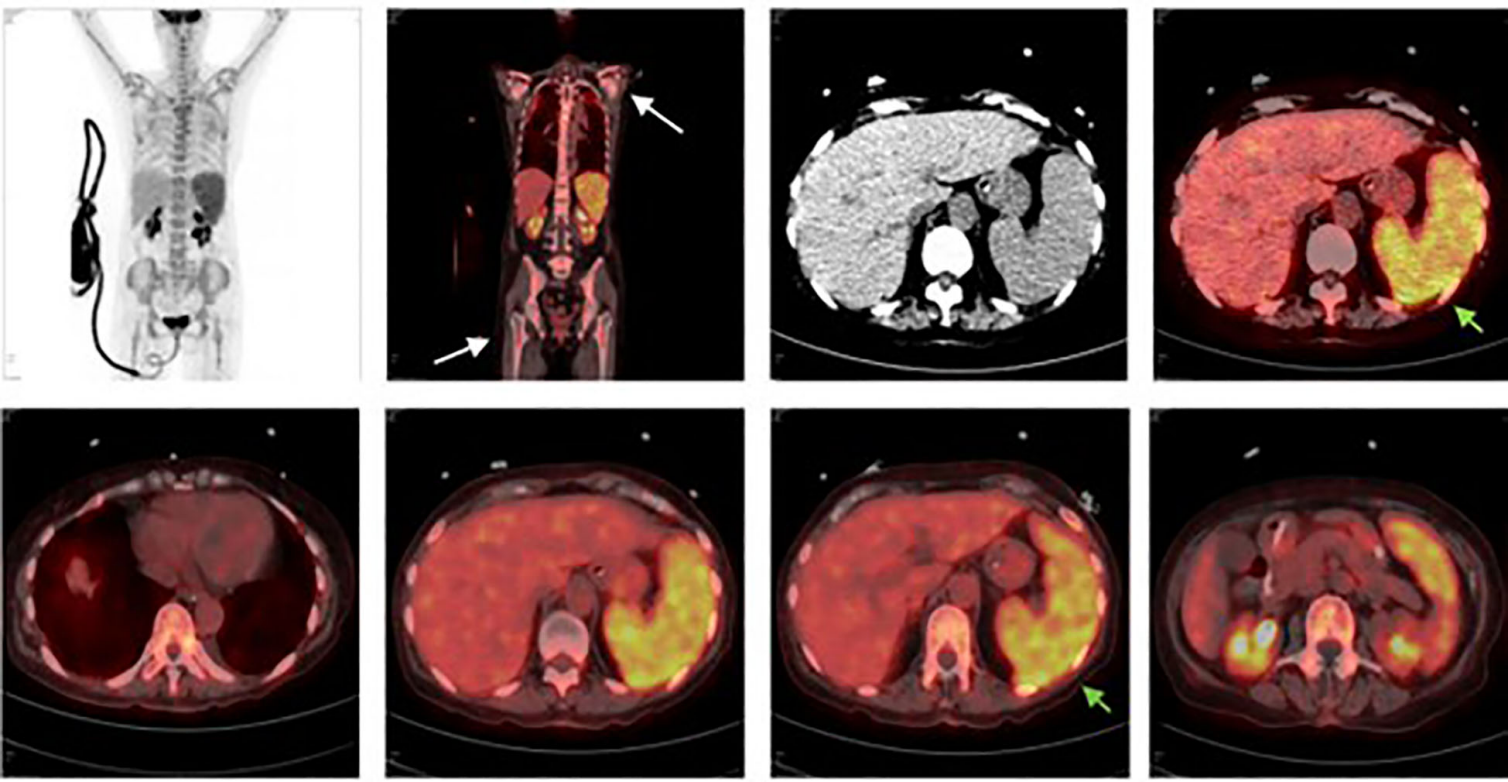
Positron Emission Tomography-Computed Tomography Findings PET/CT demonstrated splenomegaly with diffusely increased FDG uptake (SUVmax 4.74) and mild diffuse hypermetabolism in the bone marrow (SUVmax 4.06). Diffuse increased marrow uptake was also observed in the bilateral patellae, femora, and tibia-fibulae, with the most prominent lesion located in the left patella (SUVmax 5.31). These findings raised suspicion for occult hematologic malignancy and supported repeat bone marrow evaluation.

To further elucidate the underlying etiology, PET/CT was performed. Imaging revealed splenomegaly with diffusely increased FDG uptake (SUVmax 4.74) and mild diffuse hypermetabolism in the bone marrow (SUVmax 4.06). In addition, diffuse increased marrow uptake was noted in the bilateral patellae, femora, and tibia-fibulae, with the most prominent uptake in the left patella (SUVmax 5.31) (**Figure 3**).

These findings raised strong suspicion for an underlying hematologic malignancy.

Based on these imaging abnormalities, a repeat unilateral bone marrow biopsy was subsequently performed at the right posterior superior iliac spine under PET-CT guidance. Histopathological examination demonstrated sheets of large atypical lymphoid cells expressing CD20 and CD79a, consistent with diffuse large B-cell lymphoma (non-germinal center B-cell type) involving the bone marrow. Molecular analysis further confirmed B-cell clonality. Taken together, these findings established the diagnosis of diffuse large B-cell lymphoma involving the bone marrow and were reviewed by the pathology department (**Table 3** and **Supplementary Figure 1**).

PET/CT demonstrated splenomegaly with diffusely increased FDG uptake (SUVmax 4.74) and mild diffuse hypermetabolism in the bone marrow (SUVmax 4.06). Diffuse increased marrow uptake was also observed in the bilateral patellae, femora, and tibia-fibulae, with the most prominent lesion located in the left patella (SUVmax 5.31). These findings raised suspicion for occult hematologic malignancy and supported repeat bone marrow evaluation.

**Figure 4:**
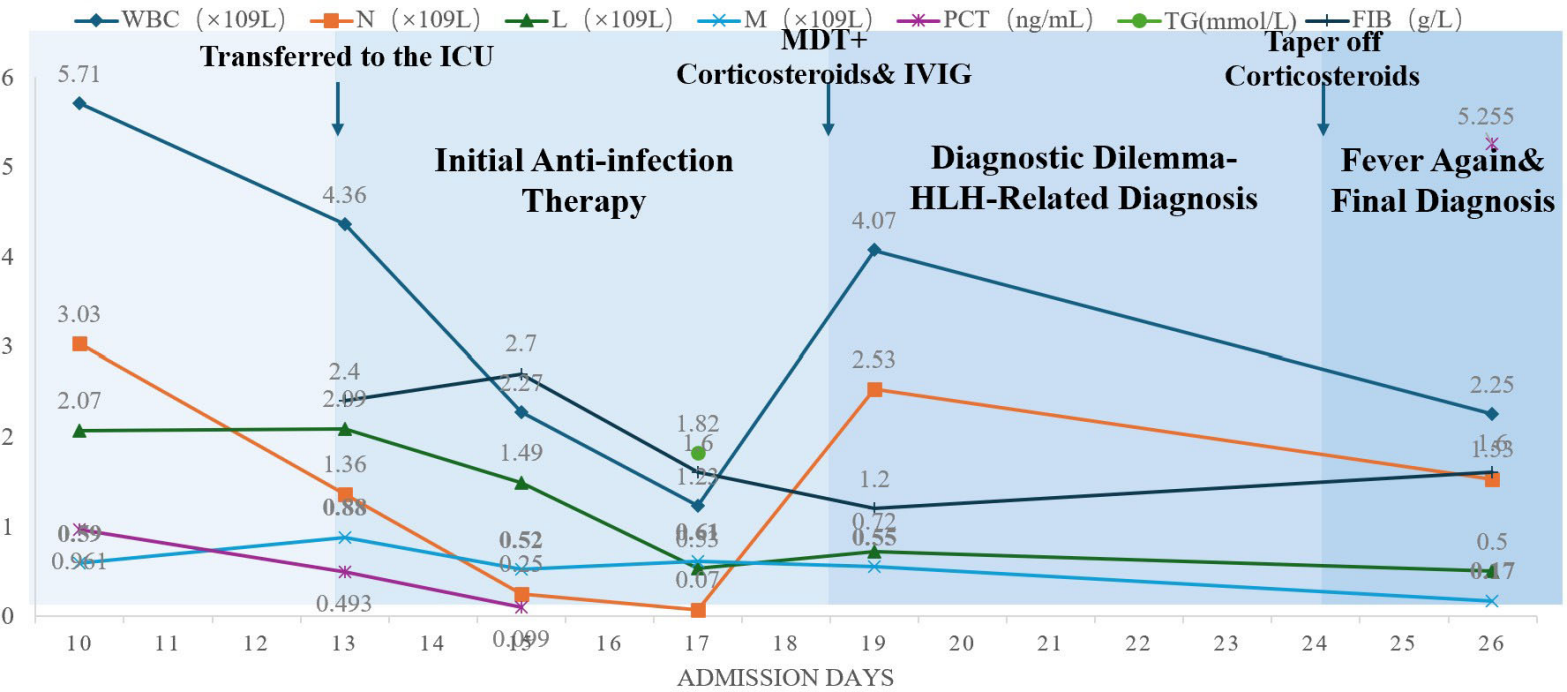
Temporal Trends of Key Laboratory Parameters Dynamic changes in inflammatory and hematologic markers during hospitalization, including leukocyte counts, ferritin, fibrinogen, and soluble interleukin-2 receptor levels, illustrating disease progression, transient response to corticosteroids, and subsequent diagnostic clarification following PET/CT-guided repeat bone marrow biopsy.

The patient was treated with polatuzumab vedotin, ibrutinib, and rituximab. Fever resolved rapidly following initiation of therapy, and clinical condition improved substantially. Follow-up bone marrow examination showed active trilineage hematopoiesis without diffuse tumor infiltration, indicating favorable hematologic response. (**Tables 3**, **4** and **Figure 4**).

**Table 4:** Clinical course timeline.

Time	Clinical event	Key findings	Clinical decision
Day 0	Admission with fever	CT: Infection	Initiate anti-infection therapy
Day 13-18	Persistent high fever	CBC: Decreased WBC, Ferritin: Elevated	HLH considered
Day 18-19	MDT+ Bone Marrow	–	Add corticosteroids and immunoglobulin therapy
Day 20-24	Initial bone marrow biopsy negative	No tumor cells detected	Taper off Corticosteroids
Day 25	Recurrent fever after steroid discontinuation, PET/CT	Diffuse uptake in spleen and bone marrow	Repeat bone marrow biopsy
Day 28	Second bone marrow biopsy → DLBCL	Non-GCB subtype	Initiate Polatuzumab vedotin + Ibrutinib + Rituximab regimen
Day 40-150	Positive treatment response+ Bone Marrow	Symptoms stable	–

Dynamic changes in inflammatory and hematologic markers during hospitalization, including leukocyte counts, ferritin, fibrinogen, and soluble interleukin-2 receptor levels, illustrating disease progression, transient response to corticosteroids, and subsequent diagnostic clarification following PET/CT-guided repeat bone marrow biopsy.

## 3 Discussion

### 3.1 Atypical symptoms: tongue pain and extranodal involvement

The tongue possesses a complex lymphatic drainage system, with lymphatic flow primarily directed toward the submental, submandibular, and deep cervical lymph nodes. In addition, several anatomical studies have described intermediate lymphatic pathways located between the lingual mucosa and regional lymph nodes. Although most evidence regarding these pathways originates from investigations of oral malignancies, their anatomical characteristics suggest that the submucosal and muscular layers of the tongue may potentially serve as sites of extranodal involvement even in the absence of overt mass lesions or clinically detectable lymphadenopathy.

Previous reports have described that primary or secondary lingual lymphoma may present not only with focal swelling or ulceration but also with neuropathic pain or sensory disturbances (6, 12, 13). These manifestations are thought to be related to tumor infiltration, local inflammatory responses, or possible perineural involvement. When lymphoma cells infiltrate the submucosal tissues, muscular layers, or adjacent neural structures of the tongue, patients may experience early, nonspecific symptoms such as pain or discomfort, which may precede the appearance of detectable structural abnormalities.

DLBCL most commonly presents with lymphadenopathy and systemic symptoms such as fever, whereas lingual involvement is rarely reported. In the present case, tongue pain appeared early in the disease course and persisted throughout disease progression, showing a temporal pattern that paralleled the patient’s systemic inflammatory activity and fever. Notably, no discrete lingual mass or significant cervical lymphadenopathy was identified during clinical evaluation, making the symptom initially more likely to be attributed to infection or nonspecific inflammatory conditions.

Furthermore, the coexistence of hemophagocytic lymphohistiocytosis (HLH) further complicated the diagnostic process. HLH-related manifestations, including persistent fever, cytopenias, and severe systemic inflammation, often direct clinical attention toward infectious or immune-mediated etiologies, potentially masking the presence of an underlying malignancy. In this context, the persistent tongue pain observed in our patient may represent a nonspecific but clinically relevant symptom that preceded the eventual diagnosis of lymphoma.

Therefore, although tongue pain cannot be considered a specific manifestation of lymphoma, this case suggests that persistent oral or lingual discomfort of unclear etiology, particularly when accompanied by systemic inflammatory features or HLH-like presentations, may warrant careful evaluation for underlying hematologic malignancies.

### 3.2 DLBCL with bone marrow involvement

Bone marrow biopsy provides essential histopathological information that plays a critical role in the staging and management of diffuse large B-cell lymphoma. Bone marrow involvement has been reported in approximately 10–30% of patients with DLBCL and is associated with important prognostic and therapeutic implications (14). However, bone marrow infiltration in DLBCL is frequently focal or patchy rather than diffusely distributed (15). Consequently, single-site bone marrow biopsy may be subject to sampling bias and may fail to detect tumor infiltration, resulting in false-negative results during the initial evaluation. Several studies have demonstrated that repeat or bilateral bone marrow biopsies can significantly improve the detection rate of marrow involvement (11, 16, 17).

In the present case, the initial bone marrow examination revealed active trilineage hematopoiesis without evidence of malignant infiltration. Nevertheless, PET/CT subsequently demonstrated diffuse hypermetabolic activity within the bone marrow and abnormal uptake in multiple skeletal sites, raising strong suspicion for an underlying hematologic malignancy. Guided by these imaging findings, repeat bone marrow biopsy was performed and ultimately confirmed DLBCL with marrow involvement.

This case therefore highlights the potential limitations of a single bone marrow biopsy in detecting focal marrow infiltration in DLBCL. When clinical suspicion remains high, particularly in the presence of unexplained systemic inflammation, HLH, or abnormal metabolic findings on PET/CT, repeat bone marrow evaluation should be considered to reduce the risk of missed diagnosis.

### 3.3 The role of PET/CT in diagnosing DLBCL with bone marrow involvement

Bone marrow involvement in DLBCL frequently demonstrates a focal or patchy distribution, which may lead to false-negative findings on initial biopsy due to sampling error. This limitation underscores the importance of repeat bone marrow examination or image-guided biopsy when clinical suspicion for lymphoma remains high.

As a noninvasive imaging modality, 18F-FDG PET/CT can reveal metabolic disease patterns that may not be detected by conventional diagnostic approaches. Several studies have demonstrated that FDG PET/CT provides high diagnostic performance for detecting bone marrow involvement in DLBCL. A systematic review reported that PET/CT achieved a sensitivity of approximately 77%, specificity of about 92%, and a negative predictive value approaching 97% in identifying marrow infiltration in DLBCL patients. In comparison, the sensitivity of routine bone marrow biopsy was considerably lower due to the focal or patchy nature of marrow involvement. These findings highlight the complementary role of PET/CT and pathological evaluation, particularly when initial bone marrow biopsy results are inconclusive (10, 18–22).

In addition to its diagnostic role, PET/CT provides important information for disease evaluation and treatment monitoring. Increasing evidence suggests that PET/CT can characterize tumor heterogeneity, while baseline metabolic parameters such as metabolic tumor volume (MTV) and total lesion glycolysis (TLG) measured before immunochemotherapy may predict treatment response and stratify patients according to survival risk (23, 24). These findings highlight the importance of integrating imaging findings with clinical and laboratory data when evaluating suspected bone marrow involvement in lymphoma.

### 3.4 HLH: a diagnostic blind spot in B-cell lymphoma

Persistent fever that responds poorly to antimicrobial therapy is a hallmark manifestation of HLH. HLH is most commonly triggered by infections, malignancies, autoimmune disorders, or immunodeficiency states, and its pathophysiology reflects cytokine-driven immune dysregulation rather than uncontrolled infection alone.

In LAHS, excessive activation of cytotoxic T lymphocytes and macrophages results in sustained release of pro-inflammatory cytokines such as interferon-γ, interleukin-6, and tumor necrosis factor-α. In some cases of B-cell lymphoma-associated HLH, this immune activation may precede overt morphologic evidence of malignancy, thereby delaying recognition of the underlying lymphoma.

Compared with HLH associated with T- or NK-cell lymphomas, B-cell LAHS is often clinically subtle. Patients may present with relatively low tumor burden, minimal lymphadenopathy, and nonspecific imaging findings, allowing immune-mediated systemic inflammation to dominate the early clinical course. Under these circumstances, conventional imaging and initial pathological sampling may fail to detect malignant infiltration, increasing the risk of delayed diagnosis (9, 25–28).

In the present case, HLH-related clinical and laboratory abnormalities, including persistent fever, progressive cytopenias, and markedly elevated ferritin levels, were evident before pathological confirmation of DLBCL, suggesting that immune dysregulation predominated during the early disease phase. This process likely contributed to the initially nondiagnostic bone marrow biopsy by obscuring the underlying lymphoma.

Therefore, in patients presenting with fever of unknown origin accompanied by HLH features, particularly after infectious causes have been excluded, clinicians should maintain a high index of suspicion for occult lymphoma. Early use of functional imaging modalities such as PET/CT to guide repeat and targeted tissue sampling may facilitate timely diagnosis.

## 4 Conclusion

This case highlights the diagnostic complexity of DLBCL presenting with HLH and atypical clinical manifestations. HLH may precede overt morphologic evidence of malignancy and may transiently respond to corticosteroid therapy, thereby masking the underlying disease. Importantly, a single negative bone marrow biopsy does not exclude lymphoma, particularly when marrow involvement is patchy.

Persistent fever refractory to antimicrobial therapy, together with unexplained cytopenias and elevated inflammatory markers, should prompt early consideration of occult hematologic malignancy. Functional imaging with PET/CT plays a crucial role in identifying metabolically active lesions and guiding repeat targeted tissue sampling. Recognition of atypical clinical manifestations, combined with early PET/CT-guided reassessment, may reduce diagnostic delay and allow timely initiation of disease-specific therapy in critically ill patients.

## Data Availability

The original contributions presented in the study are included in the article/**Supplementary Material**. Further inquiries can be directed to the corresponding author.

## Glossary

B-cell LAHS: B-cell Lymphoma-Associated Hemophagocytic Lymphohistiocytosis
CBC: Complete Blood Count
CNS: Central Nervous System
CRP: C-Reactive Protein
CT: Computed Tomography
DLBCL: Diffuse Large B-cell Lymphoma
EB: Epstein-Barr virus
ECG: Electrocardiogram
EF: Ejection Fraction
FDG: Fluorodeoxyglucose
FER: Ferritin
FIB: Fibrinogen
HLH: Hemophagocytic Lymphohistiocytosis
LAHS: Lymphoma-Associated Hemophagocytic Syndrome
MDT: Multidisciplinary Team
MTV: Metabolic Tumor Volume
NK: Natural Killer (cells)
NHL: Non-Hodgkin Lymphoma
PCT: Procalcitonin
PET/CT: Positron Emission Tomography-Computed Tomography
PCR: Polymerase Chain Reaction
sIL-2R/sCD25: Soluble Interleukin-2 Receptor
SV: Stroke Volume
TG: Triglycerides
TLG: Total Lesion Glycolysis

## References

[1] Lopez-Santillan M Lopez-Lopez E Alvarez-Gonzalez P Martinez G Arzuaga-Mendez J Ruiz-Diaz I . Prognostic and therapeutic value of somatic mutations in diffuse large B-cell lymphoma: a systematic review. Crit Rev Oncol Hematol. (2021) :165:103430. doi: 10.1016/j.critrevonc.2021.103430, PMID: 34339834

[2] Zhang QY Foucar K . Bone marrow involvement by hodgkin and non-hodgkin lymphomas. Hematol Oncol Clin North Am. (2009) 23:873–902. doi: 10.1016/j.hoc.2009.04.014, PMID: 19577173

[3] Hmidi M Touiheme N Elboukhari A Kettani M Elmejareb C Messary A . Primary B cell lymphoma of the tongue: a case report. Pan Afr Med J. (2012) 12. doi: 10.11604/pamj.2012.12.5.668, PMID: 22826730 PMC3396871

[4] Silva TDB Ferreira CBT Leite GB Pontes JR de M Antunes HS . Oral manifestations of lymphoma: a systematic review. (2016) 12:5. doi: 10.3332/ecancer.2016.665, PMID: 27594910 PMC4990057

[5] Kemp S Gallagher G Kabani S Noonan V O’Hara C . Oral non-Hodgkin’s lymphoma: review of the literature and World Health Organization classification with reference to 40 cases. Oral Surg Oral Med Oral Pathol Oral Radiol Endod. (2008) 105:194–201. doi: 10.1016/j.tripleo.2007.02.019, PMID: 17604660

[6] Arifin AJ Lam S MacNeil SD . A case report of a primary lymphoma of the tongue presenting as trigeminal neuralgia. J Otolaryngol Head Neck Surg J Oto Rhino Laryngol Chir Cervico Faciale. (2019) 48:37. doi: 10.1186/s40463-019-0360-9, PMID: 31383004 PMC6683536

[7] Shakoory B Geerlinks A Wilejto M Kernan K Hines M Romano M . The 2022 EULAR/ACR points to consider at the early stages of diagnosis and management of suspected haemophagocytic lymphohistiocytosis/macrophage activation syndrome (HLH/MAS). Ann Rheum Dis. (2023) 82:1271–85. doi: 10.1136/ard-2023-224123, PMID: 37487610 PMC11017727

[8] Atteritano M David A Bagnato G Beninati C Frisina A Iaria C . Haemophagocytic syndrome in rheumatic patients. A systematic review. Eur Rev Med Pharmacol Sci. (2012) 16:1414–24. 23104659

[9] Han AR Lee HR Park BB Hwang IG Park S Lee SC . Lymphoma-associated hemophagocytic syndrome: clinical features and treatment outcome. Ann Hematol. (2007) 86:493–8. doi: 10.1007/s00277-007-0278-6, PMID: 17347847

[10] Doma A Zevnik K Studen A Prevodnik VK Gasljevic G Novakovic BJ . Detection performance and prognostic value of initial bone marrow involvement in diffuse large B-cell lymphoma: a single centre 18F-FDG PET/CT and bone marrow biopsy evaluation study. Radiol Oncol. (2024) 58:15–22. doi: 10.2478/raon-2024-0004, PMID: 38378029 PMC10878769

[11] Kim MS Cho YU Jang S Seo EJ Lee JH Park CJ . A case of primary bone marrow diffuse large B-cell lymphoma presenting with fibrillar projections and hemophagocytic lymphohistiocytosis. Ann Lab Med. (2017) 37:544–6. doi: 10.3343/alm.2017.37.6.544, PMID: 28840996 PMC5587831

[12] Gvetadze SR Ilkaev KD . Lingual lymph nodes: anatomy, clinical considerations, and oncological significance. World J Clin Oncol. (2020) 11:337–47. doi: 10.5306/wjco.v11.i6.337, PMID: 32874949 PMC7450815

[13] Ghosh R Pandey S Sinha B Gupta RK Dandekar M . Lingual lymph nodes in tongue cancers: a systematic review and meta-analysis. Br J Oral Maxillofac Surg. (2025) 63:554–61. doi: 10.1016/j.bjoms.2025.05.017, PMID: 40754475

[14] Friedberg JW . Relapsed/refractory diffuse large B-cell lymphoma. Hematol Am Soc Hematol Educ Program. (2011) 2011:498–505. doi: 10.1182/asheducation-2011.1.498, PMID: 22160081

[15] Park SH Lee EY Chung JS . A rare case of diffuse large B cell lymphoma-associated hemophagocytic syndrome initially present in the bone marrow with a favorable clinical course. Blood Res. (2016) 51:144–7. doi: 10.5045/br.2016.51.2.144, PMID: 27382563 PMC4931936

[16] Brunning RD Bloomfield CD McKenna RW Peterson LA . Bilateral trephine bone marrow biopsies in lymphoma and other neoplastic diseases. Ann Intern Med. (1975) 82:365–6. doi: 10.7326/0003-4819-82-3-365, PMID: 1172924

[17] Wang J Weiss LM Chang KL Slovak ML Gaal K Forman SJ . Diagnostic utility of bilateral bone marrow examination: significance of morphologic and ancillary technique study in Malignancy. Cancer. (2002) 94:1522–31. doi: 10.1002/cncr.10364, PMID: 11920510

[18] Badr S Kotb M Elahmadawy MA Moustafa H . Predictive value of FDG PET/CT versus bone marrow biopsy in pediatric lymphoma. Clin Nucl Med. (2018) 43:e428–38. doi: 10.1097/RLU.0000000000002315, PMID: 30358625

[19] Al-Ibraheem A Abdlkadir AS Hasasna N Alalawi H Mohamedkhair A Al-Yazjeen S . The role of [18F]FDG PET and clinicopathologic factors in detecting and predicting bone marrow involvement in non-hodgkin lymphoma. Cancers. (2025) 17:231. doi: 10.3390/cancers17020231, PMID: 39858013 PMC11763818

[20] McCarten KM Nadel HR Shulkin BL Cho SY . Imaging for diagnosis, staging and response assessment of hodgkin lymphoma and non-hodgkin lymphoma. Pediatr Radiol. (2019) 49:1545–64. doi: 10.1007/s00247-019-04529-8, PMID: 31620854

[21] Alyamany R El Fakih R Alnughmush A Albabtain A Kharfan-Dabaja MA Aljurf M . A comprehensive review of the role of bone marrow biopsy and PET-CT in the evaluation of bone marrow involvement in adults newly diagnosed with DLBCL. Front Oncol. (2024) 14:1301979 PubMed PMID: 38577334. doi: 10.3389/fonc.2024.1301979, PMID: 38577334 PMC10991722

[22] Almaimani J Tsoumpas C Feltbower R Polycarpou I . FDG PET/CT versus bone marrow biopsy for diagnosis of bone marrow involvement in non-hodgkin lymphoma: a systematic review. Appl Sci. (2022) 12:540. doi: 10.3390/app12020540, PMID: 41725453

[23] Prieto Prieto JC Vallejo Casas JA Hatzimichael E Fotopoulos A Kiortsis DN Sioka C . The contribution of metabolic parameters of FDG PET/CT prior and during therapy of adult patients with lymphomas. Ann Nucl Med. (2020) 34:707–17. doi: 10.1007/s12149-020-01521-3, PMID: 32924071

[24] Filippi L Ferrari C Nuvoli S Bianconi F Donner D Marongiu A . Pet-radiomics in lymphoma and multiple myeloma: update of current literature. Clin Transl Imaging. (2024) 12:119–35. doi: 10.1007/s40336-023-00604-1, PMID: 41940407

[25] Ojo AS Asemota J Ojukwu S Rajeh A Bot A Smith CJ . B-cell lymphoma-associated hemophagocytic lymphohistiocytosis: a case report. Oncol Lett. (2022) 24:246. doi: 10.3892/ol.2022.13365, PMID: 35761945 PMC9214690

[26] Patel A Vakiti A Chilkulwar A Mewawalla P . Hemophagocytic lymphohistiocytosis secondary to bone marrow only B-cell lymphoma: a very rare entity with an even rarer presentation. J Hematol. (2017) 6:49–51. doi: 10.14740/jh324w, PMID: 32300392 PMC7155825

[27] El-Mallawany NK Curry CV Allen CE . Haemophagocytic lymphohistiocytosis and epstein-barr virus: a complex relationship with diverse origins, expression and outcomes. Br J Haematol. (2022) 196:31–44. doi: 10.1111/bjh.17638, PMID: 34169507

[28] Marcondes T de SP Chiattone CS Gaiolla RD . Lymphoma-associated hemophagocytic lymphohistiocytosis. Hematol Transfus Cell Ther. (2025) 48:106087. doi: 10.1016/j.htct.2025.106087, PMID: 41260042 PMC12666448

