# Supplementary material for: Case Report: A diagnostic pitfall of diffuse large B-cell lymphoma with hemophagocytic lymphohistiocytosis and atypical tongue pain: the critical role of PET/CT-guided repeat bone marrow biopsy
Source: Front Oncol. 2026 Apr 10;16:1804997. doi: 10.3389/fonc.2026.1804997 (PMC13106055; doi:10.3389/fonc.2026.1804997)
Supplement: Supplementary file 1 [file Table1.docx]

**Supplementary:**

**Table: HLH-2004 Diagnostic Criteria**

| The diagnosis of HLH can be established if Criterion 1 or 2 is fulfilled. | Patient |
| --- | --- |
| 1. A molecular diagnosis consistent with HLH: pathologic mutations of PRF1, UNC13D, STX11, STXBP2, Rab27a, LYST, SH2D1A, BIRC4, ITK, AP3β1, MAGT1, CD27 |  |
| 2.Diagnostic criteria for HLH fulfilled (5 of the 8 criteria below) |  |
| (1) Fever ≥ 38.5℃ | ✔ |
| (2)Splenomegaly |  |
| (3) Cytopenias (affecting ≥ 2 of 3 lineages in the peripheral blood) | ✔ |
| Hemoglobin < 9 g/dL (hemoglobin < 10g/dL in infants < 4 weeks) | ✔ |
| Platelets < 100×10^9^/mL |  |
| Neutrophils < 1× 10^9^/ml | ✔ |
| (4)Hypertriglyceridemia and/or hypofibrinogenemia | ✔ |
| Fasting triglycerides ≥ 3.0 mmol/L (ie, ≥265 mg/dL) |  |
| Fibrinogen ≤ 1.5 g/L | ✔ |
| (5)Hemophagocytosis in bone marrow or spleen or lymph nodes.  No evidence of malignancy. | |
| (6)Low or no NK cell activity (according to local laboratory reference) |  |
| (7)Ferritin ≥ 500 μg/L | ✔ |
| (8)sCD25 (ie, soluble IL-2 receptor) ≥ 2400 U/mL | ✔ |
