# Supplementary material for: Case Report: A diagnostic pitfall of diffuse large B-cell lymphoma with hemophagocytic lymphohistiocytosis and atypical tongue pain: the critical role of PET/CT-guided repeat bone marrow biopsy
Source: Front Oncol. 2026 Apr 10;16:1804997. doi: 10.3389/fonc.2026.1804997 (PMC13106055; doi:10.3389/fonc.2026.1804997)
Supplement: Supplementary file 2 [file DataSheet1.docx]

**Supplementary:**


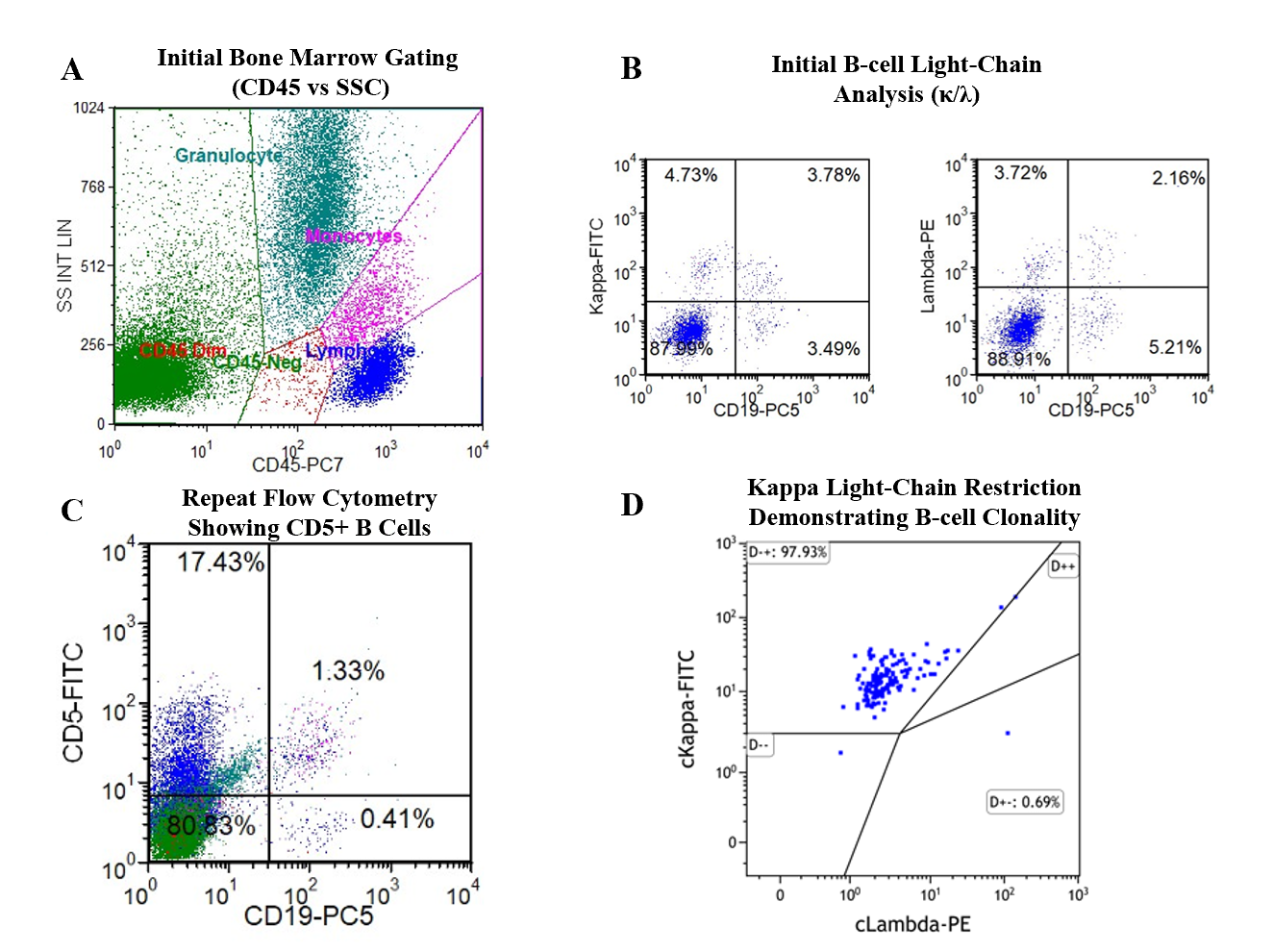


**Figure. Longitudinal Bone Marrow Flow Cytometry Findings.**

(A) Initial bone marrow flow cytometry gating based on CD45 versus side scatter (SSC), demonstrating the normal distribution of granulocytes, monocytes, and lymphocytes.

(B) Initial B-cell light-chain analysis showing a polyclonal pattern of κ and λ light-chain expression among CD19⁺ B cells.

(C) Repeat bone marrow flow cytometry revealing an abnormal CD5⁺ B-cell population within the CD19⁺ B-cell gate.

(D) Light-chain analysis demonstrating κ light-chain restriction, consistent with clonal B-cell proliferation.
